# Pasteurella multocida Bacteremia in an Immunocompromised Patient After Multiple Cat Scratches

**DOI:** 10.7759/cureus.12938

**Published:** 2021-01-27

**Authors:** Charles Boadu, Andrea Hernandez, Bassem Zeidan, Jordan T Young, Johnathan Frunzi

**Affiliations:** 1 Internal Medicine, Medical Center of Trinity, Trinity, USA

**Keywords:** immunocompromised, pasteurella multocida, sepsis, bacteremia, cat scratch, zoonosis

## Abstract

*Pasteurella multocida *(P. multocida) is a zoonotic organism found in the normal flora of domestic and wild animals. In this case report, we present a 70-year-old Caucasian male who presented with fever, chills, and greenish sputum which began hours prior to presentation. His symptoms were initially thought to be due to refractory pneumonia because the patient had been discharged from our hospital three weeks prior to presentation. Blood cultures grew *P. multocida*, a rare pathogen to cause bacteremia. He was treated with cefepime and later amoxicillin/clavulanic acid and made an uneventful recovery. Later history of the patient’s cats scratching him was thought to be the mode of transmission. *P. multocida* infection is rare, and can also be dangerous and even fatal in immunocompromised individuals. Clinicians must therefore operate with a high degree of suspicion especially when treating immunocompromised patients with bacteremia.

## Introduction

*Pasteurella multocida* (*P. multocida*) is a zoonotic organism which can be seen in patients after a bite or scratch from domestic animals such as dogs, cats, as well as wild animals such as rabbits, opossums, boars, tigers, lions and horses [1,2]. Although transmission of* P. multocida* occurs after a traumatic encounter, non-traumatic transmission from the saliva of a host has been documented [3]. Risk factors for developing *P. multocida*bacteremia include old age, a history of chronic pulmonary disease or diabetes, an immune-compromised state, and finally exposure to pet cats or dogs [3]. *P. multocida* bacteremia/septicemia is generally seen in immunocompromised patients, however, infections in immunocompetent patients have also been documented and are just as dangerous if not treated appropriately. Non-bite infections of* P. multocida* are more likely to require care in the intensive care unit (ICU), likely to occur in patients with severe comorbidities, and also likely to end in mortality [4,5]. In this case report, we present *P. multocida* bacteremia/septicemia in an immunocompromised adult male patient with end stage kidney disease and a failed kidney transplant.

## Case presentation

A 70-year-old Caucasian male initially presented to the emergency department (ED) of our hospital with fever and chills, which began one hour prior to his presentation. He also reported nausea and a productive cough with greenish sputum. The patient had been previously admitted to our hospital for pneumonia a month prior to his presentation and was discharged to a rehabilitation facility for three weeks. His past medical history was significant for end stage renal disease (ESRD) with dialysis dependence, failed kidney transplant, coronary artery disease (CAD) status with four drug-eluting stents (DES), type 2 diabetes mellitus, chronic pneumonia, and hypertension.

On admission, his vital signs were as follow: temperature 39.4°C, blood pressure 87/55 mm Hg, pulse 100 beats per minute (bpm), oxygen saturation 88% on room air and respiratory rate 18 breaths per minute. Physical examination revealed coarse breath sounds bilaterally on auscultation, a left upper arm arteriovenous fistula as well as multiple superficial abrasions bilaterally on his lower extremities. His laboratory findings were as follows: white blood cells 15.12 K/µL, platelets 104 K/µL, sodium 134 mmol/L, blood urea nitrogen 39 mg/dl, creatinine 4.5 mg/dl, lactic acid 2.77 mmol/L and procalcitonin 25.24 ng/ml. Urine analysis was not performed because the patient was anuric. His chest X-ray showed evidence of small effusions and bibasilar airspace disease which were indicative of atelectasis or infection. An electrocardiogram showed normal sinus rhythm, a rate of 99 bpm, normal axis and no significant ST abnormalities, with paroxysmal ventricular contractions (PVCs). Blood cultures were drawn, and the patient received ceftriaxone and piperacillin/tazobactam empiric antibiotics and intravenous (IV) fluids. Gram stain of the blood specimen showed gram-negative rods.

He was initially admitted to the progressive care unit (PCU) with cardiac telemetry. He was found to have second-degree atrioventricular (AV) block and was subsequently transferred to the intensive care unit (ICU) for closer management. On the second day of admission, blood cultures grew *Pasteurella multocida*. The organism was sensitive to beta-lactams including ampicillin, amoxicillin, amoxicillin/clavulanic acid and ampicillin/sulbactam. The patient was treated with IV cefepime due to his comorbidities. At this time, the patient was further questioned about recent animal contact. He stated that he lived with his wife and 14 cats and admitted the cats usually scratched him when they laid on him. His subsequent hospital stay was unremarkable. He was discharged home six days after admission with amoxicillin/clavulanic acid for two weeks. The patient returned to the outpatient clinic two weeks after discharge and had made an uneventful recovery.

## Discussion

*P. multocida* is an anaerobic gram-negative, coccobacilli organism which is found in the oral and gastrointestinal flora of domestic and wild animals such as dogs, cats, foxes, opossums, tigers, horses and lions [1,2]. Cats have the highest rate of colonization (50-90%), followed by dogs (55-60%), pigs (51%), and rats (14%) [3]. Transmission is mainly through scratches and bites, with the latter being more deadly as it runs deeper into the skin [3]. Transmission from non-traumatic methods such as inhalation of pet contaminated aerosols or from pet secretions such as saliva have also been documented [3]. In Miyoshi et al., patterns of isolates taken from a patient and her pet dog were very similar as opposed to a control used in the laboratory proving that the transmission occurred through non-traumatic means [3]. In our case, transmission likely occurred from a scratch from one of the patient’s cats.

Giordano et al. conducted a 14-year retrospective review of the Medical University of South Carolina’s laboratory information system for patients with *P. multocida* infection for the 2000-2014 period [4]. Forty-four patients were identified as infected with* P. multocida*, with 25 due to an animal bite [4]. The average age was 64 years, with the majority of patients being women [4]. Patients who presented without a bite were more frequently bacteremic (37% vs 4%, p = 0.001), and presented more often to the hospital (84% vs 44%, p = 0.012) [4]. Seven of the eight patients who required ICU-based care had a non-bite related infection and four deaths occurred, all occurring in non-bite patients [4]. The authors further concluded that non-bite *P. multocida* infections were more likely to be associated with bacteremia, severe comorbidities, an immunocompromised state, and required ICU management with a high likelihood of mortality [4,5]. Our patient was immunocompromised and also had a non-bite transmission, which placed him at a higher risk for bacteremia and death. He had significant comorbidities, which included ESRD, failed kidney transplant, type 2 diabetes mellitus, CAD and hypertension, further increasing his risk of bacteremia and death.

Infrequent and unlikely complications such as meningitis, endocarditis, and septic arthritis have also been associated with *P. multocida* [6,7]. A case study by Clarke et al., treated a patient who presented with symptoms consistent with meningitis [7]. The causative organism was discovered after a lumbar puncture revealed *P. multocida* in the cerebrospinal fluid (CSF). The patient was successfully treated with IV ceftriaxone and amoxicillin [7]. It was soon disclosed that the patient had likely contracted it from her pet dog.

In our patient,* P. multocida* was initially not considered to be a source of his bacteremia/sepsis because it is simply not a common cause of sepsis in both immunocompetent and immunocompromised patients. According to Wilson and Ho, *P. multocida *infections are highly aggressive with skin or soft tissue inflammation, often manifesting within 24 hours and causing fever, pain and lymphadenopathy [8]. Our patient did not complain of pain or lymphadenopathy, and his blood cultures reflecting *P. multocida* prompted us to pay attention to the superficial scratches on his legs as the source of infection.

Infection with *P. multocida*, even in severe cases, can be treated successfully without relapse. The probability of recurrence and resistance is low. The recommended length of treatment for *P. multocida *bacteremia is 10 to 14 days with penicillin or, alternatively, a second or third generation cephalosporin, tetracycline or fluoroquinolone [9-11]. Our patient responded well to IV cefepime while he was admitted and amoxicillin/clavulanic acid upon discharge.

## Conclusions

In conclusion,* P. multocida* is associated with high mortality in immunocompromised patients with other comorbidities. As the number of cohabitation with dogs and cats has increased, it is important to consider the diagnosis of *P. multocida *bacteremia/septicemia in any patient with febrile symptoms and exposure to cats and dogs. Physicians have to be vigilant and must operate with a high degree of suspicion when treating patients with exposure to pets with and without bites or scratches and treat patients with appropriate antibiotics. While treatment for this infection is rather straightforward, it can be easily overlooked when diagnosing patients leading to serious complications and possibly death. Pet owners may find it hard to believe that their beloved pets are reservoirs for multiple strains of bacteria that have the possibility of being life-threatening. To prevent future exposure, immunocompromised patients should be advised to limit exposure to pets. We believe a thorough history and physical exam can help physicians rule in *P. multocida *as a possible cause of bacteremia, sepsis and meningitis.
